# Clinical and psychological changes associated with music listening in haemodialysis patients: A prospective study

**DOI:** 10.4102/sajpsychiatry.v32i0.2601

**Published:** 2026-01-20

**Authors:** Fatma Zehra AĞAN, Çiğdem Cindoğlu, Neriman Sila KOÇ, Ülker Fedai, Burcu Beyazgül, Ceylan Keskin, Veysel Ağan

**Affiliations:** 1 Department of Internal Medicine, Faculty of Medicine, Harran University, Şanliurfa, Turkey; 2 Department of Nephrology, Faculty of Medicine, Harran University, Şanliurfa, Turkey; 3 Department of Psychiatry, Faculty of Medicine, Harran University, Şanliurfa, Turkey; 4 Department of Public Health, Faculty of Medicine, Harran University, Şanliurfa, Turkey; 5 Department of Health Services, Faculty of Health Sciences, Harran University, Şanliurfa, Turkey

**Keywords:** chronic kidney disease, haemodialysis, music, anxiety, depression, biochemical parameters

## Abstract

**Background:**

Chronic kidney disease (CKD) is a progressive condition associated with high morbidity and mortality. Haemodialysis (HD) patients experience significant psychological and physiological stress. Non-pharmacological interventions such as music listening sessions can alleviate anxiety and depression without drug-related side effects.

**Aim:**

This study was conducted to evaluate the effects of music therapy on psychological well-being and selected biochemical parameters in HD patients.

**Setting:**

This study was conducted with 49 HD patients at the Dialysis Unit of Harran University Faculty of Medicine between May and July 2025.

**Methods:**

All patients underwent a 4-week music listening programme (12 sessions, each session consisting of 30 min of traditional music). Psychological status was assessed before and after the intervention using the Beck Anxiety and Depression Inventories. Biochemical parameters and dialysis efficiency indicators were also recorded. Data were analysed using the Wilcoxon Signed-Rank Test.

**Results:**

Significant decreases were observed in anxiety and depression scores (*p* < 0.001). Biochemical analyses showed significant changes in sodium (*p* < 0.001), calcium (*p* = 0.002), glucose (*p* = 0.024) and albumin (*p* < 0.001) levels. No significant changes were observed in dialysis efficiency indicators.

**Conclusion:**

Music listening sessions administered during HD sessions improved patients’ psychological state and affected selected biochemical parameters. This is a safe, cost-effective, complementary intervention that may increase comfort and potentially improve physiological outcomes.

**Contribution:**

This study highlights the potential of music listening sessions as an adjunct to conventional treatments in HD care.

## Introduction

Chronic kidney disease (CKD) is a progressive condition characterised by a gradual decline in kidney function, representing a significant medical, social and economic burden with high morbidity and mortality rates.^1^ Chronic kidney disease is defined by an estimated glomerular filtration rate (eGFR) of less than 60 mL/min/1.73 m^2^ for at least 3 months.^2^ The global prevalence of CKD is increasing, and it is predicted to become the fifth leading cause of death worldwide within the next 20 years.^3^

Haemodialysis (HD) is a life-sustaining treatment for patients with CKD, yet it poses numerous psychological and physical challenges. Individuals undergoing HD frequently report heightened anxiety, discomfort and stress, stemming not only from the procedure itself but also from the broader impact of living with a chronic condition. Typical complications include sleep disturbances, muscle cramps, itching and fluctuations in blood pressure and heart rate, all of which may compromise patient comfort and treatment effectiveness, ultimately diminishing quality of life and increasing the likelihood of negative health outcomes.^4^ Depression and anxiety affect approximately 50% of patients undergoing HD,^5^ correlating with lower quality of life,^6^ poor adherence to dialysis,^7^ suicidal behaviours,^8^ increased morbidity and mortality.^6^ Although medications are available to alleviate anxiety, depression and pain in patients undergoing HD, they can lead to side effects such as sleepiness, dizziness, risk of dependence and potential drug interactions. Non-pharmacological approaches, including music therapy, acupuncture and massage, have been shown to enhance health-related quality of life without exposing patients to the risks linked to prolonged medication use. Non-pharmacological interventions, such as listening to music, acupuncture and massage, have demonstrated improvements in health-related quality of life without the risks associated with long-term drug use.^9,10,11^ Among these interventions, music listening is noted for its cost-effectiveness and its potential to alleviate anxiety and depression. It has been explored as a complementary therapy in healthcare for decades, and its use in HD has attracted attention for its possible benefits in enhancing patient well-being.^12,13,14^ Recent reviews have emphasised that non-pharmacological interventions, particularly music listening sessions, can improve psychological resilience and reduce treatment-related stress among HD patients (see e.g. meta-analyses in HD settings.^4,5,6,7,8,9,10,11,12,13,14,15^ This study aimed to evaluate the multidimensional effects of music listening in patients undergoing HD. Firstly, the influence on dialysis efficacy was investigated, including potential changes in laboratory parameters (e.g. urea, creatinine, electrolyte concentrations and dialysis adequacy indicators such as Kt/V). Secondly, the effects on patients’ psychological states were assessed using measures of anxiety and depression. By simultaneously examining biochemical and psychological outcomes, this study contributes to the limited literature addressing both aspects in HD patients, providing insight into whether music listening sessions can influence not only symptomatic relief but also treatment efficacy.

## Research methods and design

This interventional study included all patients receiving routine HD at the Dialysis Unit of Harran University Faculty of Medicine Hospital. No sampling method was employed; all patients were included (*n* = 49). Data were collected face to face between May and July 2025. The data collection instrument comprised two sections. The first section gathered demographic information, including age, gender and aetiology of dialysis. The second section included the Beck Depression Inventory (BDI) and Beck Anxiety Inventory (BAI). The BDI contains 21 items: 2 for emotions, 11 for cognition, 2 for behaviour, 5 for somatic symptoms and 1 for interpersonal concerns. Each item is scored from 0 to 3, yielding a total score of 0–63. Scores were interpreted as follows: 0–9 minimal depression, 10–18 mild, 19–29 moderate and 30–63 severe.^16^ The validity and reliability of the BDI in Turkey were established by Teğin and Hisli.^17,18^ The BAI, developed by Beck et al.^19^ and validated for the Turkish population by Ulusoy et al.,^20^ assesses the frequency of anxiety symptoms. Patients completed the questionnaires and provided blood samples prior to the intervention. The music listening sessions consisted of 12 30-min sessions of traditional Turkish instrumental music (mainly ney and oud compositions) delivered via headphones during each HD session over a period of 4 consecutive weeks. The same playlist was used for all participants, and the tempo of the selected pieces ranged from 60 beats per minute to 80 beats per minute to promote relaxation. The BDI and BAI were administered 1 day before the first session and within 24 h after the final session. As no control group was included, the study aimed to examine potential associations rather than establish causal relationships between music exposure and outcome variables. Post-intervention, blood samples and questionnaires were repeated. Dialysis efficacy was evaluated by calculating Kt/V and urea reduction ratio (URR) values before and after music listening sessions. Laboratory data were obtained from hospital records. Data analysis was conducted using Statistical Package for the Social Sciences (SPSS) version 26. Normality was assessed using skewness, kurtosis and the Shapiro–Wilk test. As the data were non-parametric, medians, standard deviations, minimum and maximum values were reported. Pre- and post-intervention values were compared using the Wilcoxon Signed-Rank Test.

All stages of this research were carried out solely by the author without any external assistance. The freely available OpenAI GPT-4o tool was used in a very limited capacity. Its use was confined to minor tasks such as paragraph formatting, section separation, standardisation of reference formatting, suggesting synonyms for no more than 10 words and correcting a few typographical errors.

### Ethical considerations

Ethical approval was granted by Harran University Clinical Research Ethics Committee (Approval Number: HRÜ/25.03.55). Written informed consent was obtained from all participants. Measures to ensure confidentiality included anonymising data and secure storage. This study involved human participants and was conducted in accordance with the ethical guidelines of the Helsinki Declaration.

## Results

Of the participants, 44.9% (*n* = 22) were female. The median age of the patients was 61 ± 2.52 years (range: 20–87), and the median duration of dialysis was 48 ± 8.04 months (range: 1–192). Among the patients, 55.1% (*n* = 27) were undergoing HD via arteriovenous fistula (AVF), while the remainder were dialysed through tunnelled dialysis catheters. Regarding the aetiologies leading to dialysis, hypertension accounted for 32.7%, idiopathic causes for 22.4% and glomerulonephritis for 14.3%, with other causes and their frequencies detailed in Table 1. The changes in patients’ clinical and laboratory values before and after the music intervention were examined. Statistically significant changes were observed in sodium, calcium, glucose and albumin concentrations (*p* = 0.001, *p* = 0.002, *p* = 0.024 and *p* =0.001, respectively). Changes in other measured parameters were not statistically significant and are presented in Table 2. Patients’ anxiety and depression experiences, measured using the BAI and BDI before and after the music intervention, are presented in Table 3. The observed changes were found to be statistically significant for both measures (*p* = 0.001 for both).

**TABLE 1:** Dialysis aetiologies of the study participants.

Aetiology	*n*	%
Hypertension	16	32.7
Diabetes mellitus ± hypertension	9	18.4
Glomerulonephritis	7	14.3
Post-renal causes	3	6.0
Polycystic kidney disease	1	2.0
Others (liver failure, solitary kidney, idiopathic)	13	26.4

**Total**	**49**	**100.0**

**TABLE 2:** Changes in patients’ clinical and laboratory values before and after music intervention.

Variable	Before music intervention (Mean ± s.d.)	After music intervention (Mean ± s.d.)	*Z* *	*P*-value
Sodium (mmol/L)	138.65 ± 3.36	136.75 ± 3.10	−3.91	< 0.001
Calcium (mg/dL)	8.72 ± 0.69	8.36 ± 0.78	−3.17	0.002
Phosphorus (mg/dL)	5.04 ± 1.63	4.64 ± 1.17	−1.37	0.171
Glucose (mg/dL)	125.36 ± 65.93	117.54 ± 64.80	−2.26	0.024
Albumin (g/dL)	3.95 ± 0.31	3.77 ± 0.26	3.84	< 0.001
WBC (×10^3^/µL)	7.93 ± 3.39	7.52 ± 3.24	−0.21	0.829
Neutrophil (×10^3^/µL)	5.22 ± 2.92	4.47 ± 1.75	−1.29	0.197
Lymphocyte (×10^3^/µL)	1.66 ± 0.74	1.59 ± 0.66	−0.60	0.546
Monocyte (×10^3^/µL)	0.63 ± 0.32	0.60 ± 0.31	−0.03	0.971
Haemoglobin (g/dL)	10.83 ± 1.57	10.96 ± 1.58	−0.75	0.452
Platelet (×10^3^/µL)	216.07 ± 70.11	203.62 ± 68.09	−1.37	0.170
PTH (pg/mL)	701.98 ± 671.35	586.60 ± 552.45	−1.45	0.145
HbA1c (%)	5.84 ± 1.35	7.27 ± 2.18	−0.96	0.336
URR (%)	74.23 ± 9.95	73.11 ± 8.25	−0.61	0.542
Kt/V	1.64 ± 0.30	1.60 ± 0.25	−0.54	0.586

URR, urea reduction ratio; s.d., standard deviation.

* , Wilcoxon Signed-Rank Test *Z* Score.

**TABLE 3:** Changes in patients’ anxiety and depression levels before and after music intervention.

Variable	Before music intervention (Mean ± s.d.)	After music intervention (Mean ± s.d.)	*Z* *	*P*-value
Beck anxiety	12.88 ± 7.87	9.57 ± 6.50	−4.55	< 0.001
Beck depression	10.48 ± 8.53	6.28 ± 6.53	−4.78	< 0.001

s.d., standard deviation.

* , Wilcoxon Signed-Rank Test *Z* Score.

## Discussion

This study aimed to evaluate the associations between music listening during the HD process and patients’ psychological states as well as certain biochemical parameters. Our findings demonstrated that music listening was associated with reductions in the extent of anxiety and depression experiences and coincided with changes in some biochemical concentrations. In our study, music listening was associated with significant reductions in the extent of anxiety and depressive experiences, as measured by the Beck Anxiety and Depression Inventories (both *p* = 0.001). This effect can be explained by music’s relaxing, attention-diverting and mood-regulating properties. Additionally, music may influence the limbic system and be associated with a reduction in the release of stress hormones.^21^ Similarly, Bradt et al. reported reductions in anxiety experiences in patients who received music listening.^22^ Other studies have highlighted that music provides emotional support and enhances psychological well-being.^23^ In this context, music listening emerges as a low-cost intervention capable of supporting psychological adaptation during long and repetitive medical procedures such as HD. The changes observed in patients’ sodium, calcium, glucose and albumin concentrations following music listening should be considered as potential associations rather than direct physiological effects of the intervention. Indeed, previous studies have demonstrated that music listening may modulate the immune system and stress response, emphasising its potential role in biological processes.^24^ Meta-analyses have shown that different types of music, the method of delivery (live versus recorded) and individuals’ personal music preferences can produce measurable changes not only in psychological experiences but also in physiological parameters such as heart rate, blood pressure, cortisol concentrations and other biomarkers.

These findings collectively indicate possible associations between musical exposure and physiological regulation although causal mechanisms remain to be established. Moreover, the individualised approach emphasised in these meta-analyses represents an important factor to consider in clinical practice to support treatment effectiveness.^25,26^ Regular and personalised application of music listening may also yield more pronounced long-term effects. The observed biochemical changes should be interpreted with caution, as they may partly reflect physiological fluctuations associated with dialysis sessions, participant expectations or other contextual factors rather than direct outcomes of the music intervention.

In this study, participants listened to traditional Turkish music during HD sessions. This intervention does not constitute clinical music therapy, and its therapeutic effects were not formally established.

### Limitations of the study

This study has several limitations. Firstly, it was conducted at a single centre with a relatively small sample size (*n* = 49), which limits the generalisability of the findings. Secondly, music listening was applied using only a specific type of music, and the effects of other music genres or individually selected music could not be evaluated. Thirdly, only short-term effects were examined, and no information was obtained regarding long-term psychological or physiological outcomes. Additionally, pre- and post-music listening blood pressure measurements could not be collected, which limited the assessment of physiological responses. Furthermore, patients’ music listening habits or cultural differences were not considered. Fourthly, changes observed in biochemical parameters may not be solely attributable to music listening, as physiological fluctuations related to dialysis sessions could also have contributed. Additionally, the intervention was implemented by a medical doctor without formal training in music listening, which may limit the standardisation of the procedure. Moreover, causal inferences cannot be drawn due to the absence of a control group.

## Conclusion

In conclusion, music listening applied during HD provides beneficial effects on both psychological well-being and certain clinical parameters. Music listening represents a low-cost, safe and highly feasible complementary method in dialysis centres. Supporting such interventions is important for enhancing patient comfort and psychological well-being in clinical practice. Music reduces stress levels, modulating the autonomic nervous system and hormonal regulation, which could in turn contribute to improved physiological balance. Our study, consistent with findings in the literature, supports the psychological and physiological benefits of music listening during HD. Therefore, music listening may serve as a valuable complementary approach integrated into chronic treatment regimens to optimise patient care. Future studies should include larger sample sizes, randomised controlled designs and different musical genres to validate these findings and explore long-term outcomes.
